# “Increase of Mental Unsoundness”—A Review of Dr. Wallace’s Paper

**Published:** 1895-06

**Authors:** A. N. Denton

**Affiliations:** Late Supt. State Lunatic Asylum, Austin


For Texas Medical Journal.

### "INCREASE OF MENTAL UNSOUNDNESS"—A REVIEW OF DR. WALLACE'S PAPER.

BY A. N. DENTON, M. D., LATE SUPT. STATE LUNATIC ASYLUM, AUSTIN.

MY ATTENTION has been called to a paper by Dr. D. R. Wallace—"Reflections upon the Increase of Mental Unsoundness"—read before the State Medical Association, and published in the May number of your JOURNAL.

Near the conclusion of the paper Dr. Wallace invites friendly criticism, and I avail myself of this invitation to indulge in a brief critical review of some statements and conclusions set forth in the paper. For obvious reasons I assume the task with great reluctance, and I shall make the attempt only because I feel it to be a duty that I owe to the medical profession.

After a careful perusal of the paper, I am not surprised that its reading created a "ripple of excitement," as stated in the JOURNAL.

More than half of the thirteen pages seems devoted to an effort to bring discredit and odium upon the Christian religion. But I have no disposition to enter the lists as a defender of Christianity. As a matter of fact it needs no defense, and if it did, there are many far more capable of performing the task than myself.

Dr. Wallace can congratulate himself that, if he did not succeed in awakening a very profound interest in the matters discussed in his paper, he at least succeeded in creating profound astonishment at some of the statements made and conclusions drawn.

When I read the title to the paper, I naturally supposed that some of the more prominent and real causes of insanity would be discussed, in connection with its apparent increase; but the reader will search in vain through the entire article for such information. Several pages are devoted to a comparison between Chinese and Christian civilizations, and I do not hesitate to say that I have rarely, if ever, seen incorporated into an article of equal length, so many inaccuracies and misstatements. This is all the more astonishing, as the author declares in the first paragraph of the paper, that he has entertained the opinions set forth for years, and, of course, the inference is that he has carefully investigated and taken pains to inform himself upon the matters discussed. He says that his opinions "have not been hastily formed. They have grown up in my mind as the result of twenty years' observation and experience with the insane." The venerable author says that the ground traveled over, as to the causes of insanity, has been already "thoroughly gleaned," and in this I agree with him, and see little profit in this "threshing of old straw." The truth is the causes of the apparent increase of insanity in certain commercial centers and large sea-coast cities of the United States has been repeatedly pointed out by alienists, and as satisfactorily accounted for, as any other phenomenon in connection with the rapidly advancing civilization and progress of our race.

Well informed men who have investigated the subject see no mystery in the apparent increase of insanity at these great centers of commerce and civilization.

Certainly no one known to me, except the learned author of the paper, has attempted to show that "institutional religion" (whatever that may mean) has been a prominent factor in the production of insanity.

I feel quite sure that in the promulgation of this idea the learned author of the paper may justly lay claim to originality.

In fact, the increase of insanity is more apparent than real, as has been repeatedly shown. The author truly says that the proportion of insane to the entire population in New York and California is as much as one to two hundred and fifty, and he might have stated the case more strongly still, as the latest statistics from the census bureau show for New York one insane to every one hundred and seventy-eight inhabitants.

Now, what is the cause of this apparent or real increase of insanity in New York? Surely no one who has investigated this subject will for a moment conclude that religion, either "institutional" or other form of it, is an important factor in the production of this unfortunate condition of sanity.

Whoever has walked through Castle Garden during the period of greatest immigration from Europe, and through the great insane hospitals on Ward's and Blackwell's islands, will hardly fail to realize the true and only important cause of the increase of insanity in New York. Such an experience will convince any unbiased observer that all other causes are as nothing compared with the dumping upon our shores of the defective population of Europe.

Under a recent act of Congress this unrestricted tide to our shores of the defective classes of European countries has been partially checked, but during the two decades immediately preceding the year 1890, immigration to this country from European nations was practically without restriction; and when we consider the great advantage accruing to the countries from which these defective classes come, in being rid of their pauper, criminal and insane population, it is no wonder that we have witnessed, during the past quarter of a century, an increase of crime, pauperism and insanity at these great centers of commerce and immigration, in a far greater ratio than the increase of the entire population of the country.

Another reason for the apparent increase of insanity throughout the civilized world, observed during the last half of the present century, is the fact that statistical information relating to the increase in every civilized country, is far more elaborate and accurate than formerly. Even during the eighteenth century a great majority of the insane were not cared for and housed as at present, but were permitted to roam at large, with little or no restraint, unless the victims of the disease were violent or dangerous, and in that case they were committed to the common jails, not for their own protection or treatment, but for the protection of the sane.

Doubtless vast numbers of such unfortunates thus perished from hunger and cold, and by violence, that were never enumerated or taken account of by the government under which they lived and died.

This great and admitted difference in statistical information upon this and kindred subjects, under different governments and at different times, is in connection with unrestricted immigration, as already stated, believed to be amply sufficient to account for the apparent increase of insanity in the United States as well as in other civilized countries.

The same facts and considerations will, in a measure at least, account for the apparent immunity from insanity enjoyed by China and other semi-civilized nations.

We have no Chinese statistical information of any value in relation to the prevalence of insanity in that country, and it is therefore misleading and idle to make comparisons and base conclusions upon such apparent differences in the absence of any reliable information.

Dr. Wallace says that "insanity in China is estimated at one in five thousand." But a very interesting question arises here, as to who makes this estimate: where does it come from?

Further on he says, "These figures will strike us as the more extraordinary, when it is recollected how extensive the opium habit prevails, especially among the Chinese." "All tolerably well informed people know that the effects ot the habit have been greatly exaggerated by Christian missionaries and others."

I regret that the author forces me by this sentence to take my position with those, who, in his opinion, are not even tolerably well informed. Because, I am convinced, from information gleaned from reliable sources, that the missionaries have not only not exaggerated the evil effects of the opium habit among the Chinese, but that it is underestimated, both in China and in this country. In the opinion of this writer, there is no personal habit now prevalent with the human race, with the exception of the liquor habit, that is so prolific of mental unsoundness as this one; and yet Dr. Wallace does not even refer to it as one of the causes of insanity. He says, on page 571, that in Japan *it is said* that any form of mental impairment is seldom met with, except in those portions of the country which have been largest and most subject to foreign influence.

This is just the character of report that every thoughtful man would expect to hear from that country. In those portions of the empire of Japan as well as of China, and other semi-civilized nations that have been exempt from foreign influence, or in other words, the influence of Christian civilization and an enlightened government, little or no account or notice is ever taken of the insane, except in cases requiring it to place them under restraint for the protection of the sane; and there is little doubt but the vast majority of this class inhabiting these countries, consisting of the milder forms of insanity that in this and other civilized countries are consigned to the splendid palatial public and private institutions, built and furnished at vast expense for their care and treatment, are taken little or no notice of, and pass their lives without recognition by their government.

Even in this country of enlightenment and civilization, where organizations of charity and philanthropy exist in every city and hamlet in every State,—whose business it is to seek out cases upon which to bestow their favors, I have myself often met with cases of the milder forms of insanity among the ignorant classes whose mental unsoundness was not recognized by their friends and relatives; and if this is true in this country, how much more it must be among the barbarous and semi-civilized peoples.

I could offer much more, and strong proof in support of this hypothesis as to the apparent infrequency of insanity in barbarous and semi-civilized countries, but want of space forbids; and I pass on to a brief consideration of the subject of religion as a cause of insanity.

With profound respect for the venerable author of the paper, I must confess to a degree of astonishment at the superficial view that he takes of this subject. He seems to have left out of consideration, and forgotten, that insanity is not a myth; but must have a real, a physical cause; and with measured sentences and eloquent perorations he pursues mere shadows that have little or no agency in the causation of mental unsoundness.

It is plainly apparent that he has mistaken a mere symptom of certain forms of insanity for one of its chief causes. I am well aware of the fact that in the courts of this, and other States, witnesses, and even members of the medical profession, often testify that religion is the cause of an attack of insanity, and these opinions of witnesses constitute a part of the record in such cases, and this record is transferred to the records of the various institutions for the care of the insane; and thence pass to the annual reports of the superintendents throughout the country. But I am of opinion that such records have no weight with the vast majority of superintendents of these institutions, in determining the real causes of insanity. They regard insanity as a disease, and that the cause of it, in the vast majority of cases, is to be sought for in certain abnormal or pathological conditions of the physical system, resulting in functional disturbance of the nervous system. Of course, this disturbance must reach the brain, in order to bring about a condition of mental unsoundness, either by reflex action from some other diseased organ, or the disturbance may be central, beginning in the brain itself; but, as before stated, there must be disease, and physical deterioration or disorganization at some point.

This being admitted, it is in the highest degree illogical to assume that religion, either "institutional" or other form of it, can be an important etiological factor in the production of insanity. As before stated, such illogical assumptions are often made by superficial observers, both professional and non-professional, from the proceedings of our courts.

Such erroneous conclusions are usually drawn from the delusive declarations and hallucinations of the victims themselves; generally in cases of melancholia. But notwithstanding the frequency with which this supposed cause appears upon the records of our institutions for the care and treatment of the insane, I venture to say, that it has no foundation in truth, in ninety-nine cases in every hundred.

I admit that there may be cases in times of great religious excitement, when there is already physical and nervous instability, and when mental equilibrium is dependent upon an imperfect and badly organized brain, and weak, and imperfect physical system, in general, that such excitement may primarily result in such functional disturbance, and ultimately in organic lesion of the brain and insanity. Mental strain, and emotional excitement from other causes and under similar circumstances, may in rare instances, produce a like result. But I pass on.

On pages 576 and 577, the learned author indulges in a most beautiful and eloquent tribute to the physical and mental perfection of the Chinese or Mongolian race.

These beautiful and eloquent passages are interspersed with some lines of poetry, which I regret I have not time or space to reproduce. Briefly stated, the aim of the author is to show, that the Chinese have been brought up to their present state of physical and mental perfection by gradual stages of evolution, or as stated by Darwin, by their "environment."

He says: "The Chinese all look alike in the main. Why? They have had the same or similar surroundings, the same scenery, been thinking the same thoughts and doing the same things for thousands of years."

And again he says: "Europe during the millenium from the fifth to the fifteenth century, desolated by religious and fanatical wars and bloodshed, followed by famine and pestilence, did not double its population. *China blessed with peace and quiet under the same imperial dynasty*, doubled her population six times." This declaration is the climax to the many other illogical and erroneous statements to be found on almost every page of this remarkable paper.

It would be difficult for an expert to frame a sentence more erroneous or inaccurate than that part of the one above quoted, relating to China.

It is true that Europe, during the period mentioned, was frequently cursed with war and pestilence; but we have great and many reasons for believing that the state of the people of European countries during the period referred to, was far better than that of the Chinese. Certainly the declaration that China was "blessed with peace and quiet" during that long and memorable period in her history, will be an amazing scrap of information to every student of history.

The truth is, there was scarcely twenty consecutive years of peace and quiet during the entire period.

On the contrary, there was almost continual war in its worst form, and upon a scale and magnitude unknown in European countries.

War in its most destructive and horrid form was the rule from century to century, from within, as well as from without her own borders; and peace was the exception.

Great armies, of from three to six hundred thousand men, and even more, repeatedly marched over the territory of every province, leaving little else but ruin, desolation and death in their pathway.

Great cities were repeatedly taken by storm or siege, and so completely destroyed as to leave nothing to tell the tale of their former greatness, and the inhabitants given over to pillage and slaughter, without regard to age or sex, to satisfy the brutal passions of a savage soldiery.

It was the boast of one of the great military chieftains of the period referred to, that the destruction of cities in his pathway, and by his direction was so complete, that horsemen could ride over the former sites of such cities without danger of stumbling over inequalities or obstructions. Almost the entire Chinese empire was conquered, and a great portion laid waste by Gengis Kahn from 1208 to 1227, and hundreds of thousands, and perhaps millions of the inhabitants were either mercilessly and ruthlessly slaughtered, or perished by famine and pestilence.

Nor was this the beginning of such wars of destruction and rapine, nor did it cease at the death of this scourge of the human race. But these wars and conquests were continued by his sons and successors until the entire empire was subdued. But I need not attempt to describe in detail the almost endless succession of wars that followed each other in quick succession throughout the long period mentioned by the author, as one of "peace and quiet, and under the same imperial dynasty."

But China was not only rent and torn by wars within, as well as from without her borders, but she was cursed with the worst form of government, that while it enslaved the people, it did not even have the merit of stability. Instead of resting in ''peace and quiet under one imperial dynasty'' for a thousand years, as stated by the author, there was at least ten dynasties during the period referred to.

It is true, that China has never had, from the earliest time in her history of which we have any reliable record, but one form of government, and that the weakest and most atrocious that was ever devised or allowed to exist by the human race. This inherent feebleness is plainly apparent to every observant student of history. She is, and has been for centuries, wholly unable to defend her frontiers against even the weakest of her neighbors. She never engages in war without being worsted, and having to purchase peace with money.

And although her citizens must prostrate themselves, and crawl upon their bellies in the presence of an effete monarch, the government, in turn, is forced continually to perform the same humiliating feat in the presence of any foreign power with which it comes in conflict.

The amazing stupidity and inherent imbecility of the government of China is well shown in the war with Japan just closed. Why is it that a nation with only 40,000,000 of inhabitants, occupying a few islands off the coast of Asia, can conquer in a few months one with 400,000,000 of inhabitants occupying 4,000,000 of square miles of territory on the main land, and having every advantage in national resources and strategic points for defense?

It can not be on account of lack of personal courage with the Chinese, because the two nations belong to the same race.

It is impossible to ascribe the failure of the Chinese to successfully defend their territory to any other cause save that of inherent weakness and imbecility on the part of the government.

This governmental imbecility, this driveling dementia, reacts upon the citizens, and slowly smothers and puts out every spark of patriotic fire that should live in the breast of every citizen of every government.

The government machinery is only terrible to her own citizens. It is of that repulsive character, which, by practising the most inhuman cruelties for the slightest offenses, has cultivated the emotion of fear and dread, to the exclusion of all the nobler passions and instincts of human nature.

In brief, this is undoubtedly the main cause of China's humiliating defeat in the late war, and of being forced to prostrate herself before a nation so vastly her inferior in numbers and national resources. Dr. Wallace dwells upon the advantages to the Chinese of living under a stable and settled government, but the government of China does not continue settled on account of any inherent strength which it possesses, but it is plainly apparent to every student of history, and intelligent observer of passing events, that it could not continue to exist for even one year but for the watchful and jealous care and guardianship of European powers.

So much for the historical features of the paper, and the resulting deductions therefrom.

A few words with reference to the author's comparison of the Chinese and Aryan races, and I have done. It would seem to the average reader almost a waste of time to draw comparisons between the Aryan and Mongolian races.

Certainly, the apparent conclusions of Dr. Wallace, if seriously considered, that the latter is superior to the former, is well calculated to produce a sensation.

What an expression of amazement such an announcement should bring to the features of the great naturalists of this generation!

He does not unequivocally declare that the Mongolian is the superior race, but such conclusion may be fairly and legitimately inferred from statements and arguments covering several pages of the paper.

He says he visited the Pacific slope and looked at the Chinaman face to face, and carefully estimated his character and measured his capacity from a scientific standpoint. These are not his exact words, but the substance of them.

But first let us get at the estimate which he places upon our own people from his own standpoint, and then allow him to speak for the Chinese. He says, on page 577:

"In manners, customs and habits, ethics and government, opinions are equally unfixed and unstable. With no fixed system of philosophy, no accepted religion, our people are afloat at the mercy of the winds and waves, without chart or compass, a starless vault overhead, on a shoreless sea, the great ocean of mortal life and human destiny, the sport of chance and of every ignorant fanaticism. We have few beliefs, *hardly any convictions, and no faith.* What religion we have is wholly intellectual,—is of the head, and not of the heart.''

Now, this is a beautiful and eloquent passage, and does great credit to the learned author. It lacks but one element to make it perfect, and that is, a basis of truth to rest upon. I do not mean that the author is untruthful, far from it. But he has unconsciously allowed his poetic and soaring imagination to warp and curve his judgment aside from the straight and narrow pathway of truth.

Is it true in religion, that we have few convictions and no faith?

A single retrospective glance at the vast army of the followers of the ''lowly Nazarene'' of every denomination who have lived and died in the faith, will convince every unbiased mind that the statement is the product of the imagination. And if the statement was made to those now living, it would provoke a negative protest from the lips of millions of our people.

Is it true that there is no unity of thought and opinion on the subject of government? This government has existed for more than a century, and to-day commands the respect of not only our own citizens, but of all people in every country where we are known. It is believed to be stronger now than it ever was. Its strength lies in the patriotic devotion of its citizens.

Ours is a government of toleration in all matters of conscience or opinion, and hence there is necessarily great diversity of opinion as to certain policies of the government; but upon the great principles of a republican form of government, we are practically a united people.

Returning to the estimate placed upon the Mongolian, or Chinese race, the author says, on page 571:

''How is it that we occidentals, Christians, appear to such disadvantage when compared with these heathen Chinese?'' And again, speaking of the visit of Marco Polo to the Chinese empire in the 13th century, he says: ''Even the common people are described in China as dressing in brocade silk and broad-cloth, living in houses adorned with rich tapestry, when our ancestors were living in caves, hollow trees and mud hovels, and dressing in skins, with wisps of straw about their legs.''

So far as the above quotation refers to the condition of the people of European countries, I think every student of history must admit that it is a gross exaggeration of the facts.

On same page, he speaks of the immense gilded palaces, and porcelain towers, as evidence of the advanced civilization of the Chinese.

And again, on page 574, he says: "The Chinese coming to this country, of course, are from an inferior stratum of society. Yet I think they are the most intelligent laborers we have. When it is remembered that the Chinese we see upon our Western slope, are from the least intelligent part of the population, one is struck with their fine cranial development, their quiet immobile features and measured movements."

On the same page, the author mentions General Grant's tour around the world, and says that although he was honored with an introduction to Prince Bismarck, Lord Beaconsfield, Gambetta, and Li Hung Chang, he decided the latter to be greatest of them all

I think the author might, with great propriety, have left out the relation of this latter incident. Surely it can not be reasonably supposed that General Grant's complimentary reference to the intellectual greatness of the prime minister of China is valuable evidence in favor of the superiority of the Chinese over the Aryan race.

I have made these quotations in justice to the author of the paper. There is little doubt but most of what was told of Chinese greatness by Marco Polo was true; but we may feel very sure, in view of the history of the country, which I have already referred to, that his descriptions of the gilded palaces and porcelain towers referred to the rich and great, and not to the common people. And I may add that great public works, such as the author describes, of mediæval and ancient times, are not evidence of advanced civilization of the people, but rather of slavery, as is witnessed in ancient Egypt, where the grandest and most imposing public works were carried to completion, the massive splendor and beauty of which is the wonder of even this generation; but it is well understood that these great works could never have been accomplished except through the slavery and consequent misery of the common people.

---

**The Latest Sensation in Paris** is the alleged important discovery by a M. Groussier, of an infallible law whereby the paternity of children who have no acknowledged father may be ascertained.

